# Balancing hydrogen adsorption/desorption by orbital modulation for efficient hydrogen evolution catalysis

**DOI:** 10.1038/s41467-019-12012-z

**Published:** 2019-09-06

**Authors:** Feng Li, Gao-Feng Han, Hyuk-Jun Noh, Jong-Pil Jeon, Ishfaq Ahmad, Shanshan Chen, Changduk Yang, Yunfei Bu, Zhengping Fu, Yalin Lu, Jong-Beom Baek

**Affiliations:** 1 School of Energy and Chemical Engineering/Center for Dimension-Controllable Organic Frameworks, Ulsan National Institute of Science and Technology (UNIST), 50 UNIST, Ulsan, 44919 South Korea; 2 MOE Key Laboratory of Low-grade Energy Utilization Technologies and Systems, CQU-NUS Renewable Energy Materials & Devices Joint Laboratory, School of Energy & Power Engineering, Chongqing University, Chongqing, 400044 P. R. China; 3 Department of Energy Engineering, School of Energy and Chemical Engineering, Perovtronics Research Center, Low Dimensional Carbon Materials Center, Ulsan National Institute of Science and Technology (UNIST), 50 UNIST, Ulsan, 44919 South Korea; 4 School of Environmental Science and Engineering, Nanjing University of Information Science and Technology, 219 Ningliu, Nanjing, Jiangsu 210044 P. R. China; 5 CAS Key Laboratory of Materials for Energy Conversion, Hefei National Laboratory for Physical Sciences at Microscale, National Synchrotron Radiation Laboratory, University of Science and Technology of China, 96 Jinzhai, Hefei, Anhui 230026 P. R. China

**Keywords:** Catalyst synthesis, Catalytic mechanisms, Electrocatalysis, Nanoparticles

## Abstract

Hydrogen adsorption/desorption behavior plays a key role in hydrogen evolution reaction (HER) catalysis. The HER reaction rate is a trade-off between hydrogen adsorption and desorption on the catalyst surface. Herein, we report the rational balancing of hydrogen adsorption/desorption by orbital modulation using introduced environmental electronegative carbon/nitrogen (C/N) atoms. Theoretical calculations reveal that the empty d orbitals of iridium (Ir) sites can be reduced by interactions between the environmental electronegative C/N and Ir atoms. This balances the hydrogen adsorption/desorption around the Ir sites, accelerating the related HER process. Remarkably, by anchoring a small amount of Ir nanoparticles (7.16 wt%) in nitrogenated carbon matrixes, the resulting catalyst exhibits significantly enhanced HER performance. This includs the smallest reported overpotential at 10 mA cm^−2^ (4.5 mV), the highest mass activity at 10 mV (1.12 A mg_Ir_^−1^) and turnover frequency at 25 mV (4.21 H_2_ s^−1^) by far, outperforming Ir nanoparticles and commercial Pt/C.

## Abstract (web summary)

Hydrogen adsorption/desorption behavior plays a key role in hydrogen evolution reaction catalysis. Here, the authors demonstrate the rational balancing of hydrogen adsorption/desorption by orbital modulation for significantly enhanced hydrogen evolution performance.

## Introduction

Because of its potential value in efficient and scalable hydrogen production, water splitting via the electrochemical hydrogen evolution reaction (HER) has attracted considerable attention from industrial and scientific communities^1,2^. Even though they are the heart of hydrogen evolution catalysis, catalysts with satisfactory performance are still rare, despite the tremendous effort that has been devoted to improving HER catalysts^3–11^. Among various approaches, sulfur (S) vacancies and strain have been introduced to activate and optimize the basal plane of monolayer 2H–MoS_2_, leading to the highest intrinsic HER activity among molybdenum–sulfide-based catalysts^8^. A high concentration of strained metallic 1T WS_2_ sites, obtained by chemical exfoliation, led to WS_2_ nanosheets with enhanced catalytic activity^4^. Ultrathin Pt nanowires on single-layered Ni(OH)_2_ nanosheets with a unique hybrid nanostructure were also achieved, and exhibited unprecedented catalytic activity and stability toward HER^6^. Nonetheless, in spite of these achievements, strategic exploration to improve the HER performance of catalysts is still a great challenge.

From a fundamental viewpoint, the hydrogen adsorption/desorption behaviors of the solid surfaces of catalysts are of crucial importance in hydrogen evolution catalysis^12,13^. Stronger hydrogen adsorption enables faster proton supply for the reaction, however, on the other side, this leads to weaker hydrogen desorption and slower release of active sites. Too strong or too weak hydrogen adsorption/desorption will lead to either active site poisoning or inefficient proton supply, resulting in a sluggish hydrogen evolution reaction rate.

The hydrogen adsorption/desorption behaviors of transition metal-based catalysts depend largely on the interaction between the unfulfilled *d* orbitals of the transition metals and the *s* orbital of hydrogen^14^. As a result, hydrogen adsorption/desorption on such catalysts can be tailored by tuning the *d* orbitals of the transition metals. With their small atomic radius and high electronegativity, the *p* orbitals of carbon and nitrogen are able to efficiently interact with the *d* orbitals of the transition metals^15^. This provides an important opportunity to enhance the catalytic activity of catalysts toward HER via orbital manipulation.

Herein, we demonstrate the rational balancing of hydrogen adsorption/desorption on a solid catalyst surface to archive significantly enhanced hydrogen evolution performance via orbital modulation. Density functional theory (DFT) calculations revealed that the empty *d* orbitals of iridium (Ir) sites can be tailored by an electronegative carbon/nitrogen (C/N) environment, which will further balance hydrogen adsorption/desorption behaviors and boost the hydrogen evolution catalysis on the surficial Ir sites. Importantly, even with a low-Ir concentration of 7.16 wt%, when Ir nanoparticles (IrNP) were anchored on a hollow nitrogenated carbon matrix (IrHNC), the resulting catalyst exhibited HER performance superior to IrNP and commercial Pt/C, including much faster reaction kinetics, and much higher mass activity as well as intrinsic activity.

## Results

### DFT calculations

**Fig. 1:**
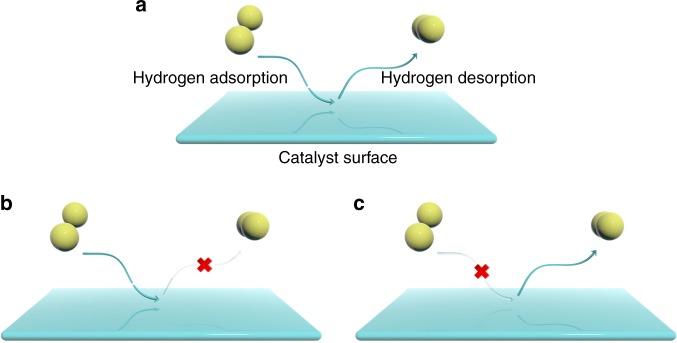
Schematic illustration for the different hydrogen adsorption/desorption behaviors of catalyst surface. **a** Balanced hydrogen adsorption/desorption behavior. **b**, **c** Too strong and too weak hydrogen adsorptions, respectively, resulting in sluggish hydrogen evolution reaction rate. The dark yellow ball represents hydrogen atom

Figure 1 shows the different hydrogen adsorption/desorption behaviors on the solid surface of catalyst. For an efficient HER, fast reactant supply and fast product delivery are needing to be satisfied simultaneously, which required strong hydrogen adsorption and strong hydrogen desorption capabilities from the catalyst surface. However, as the two sides of the coin, they cannot be achieved at the same time. Thus, the hydrogen adsorption/desorption behaviors balancing offers an alternative way to enhance the HER performance of the catalysts.

**Fig. 2:**
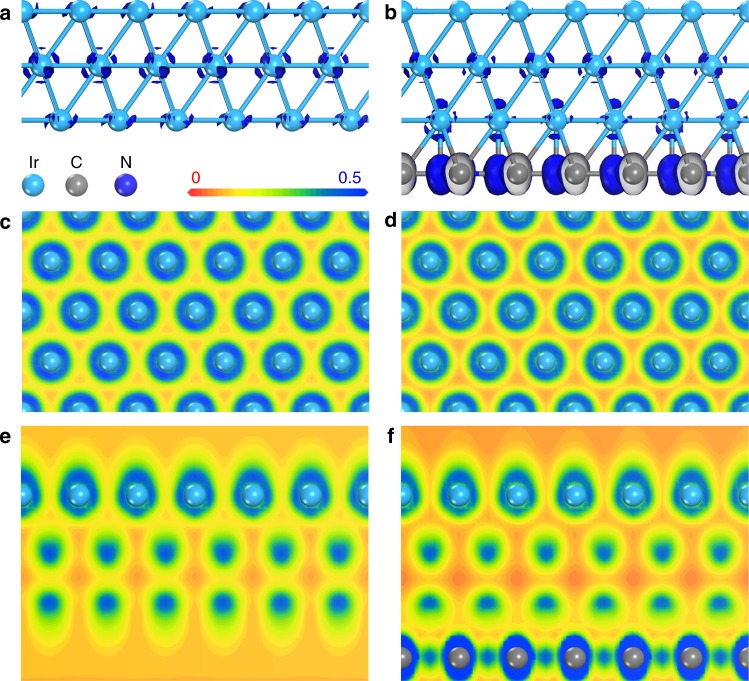
Theoretical calculations of the orbitals for Ir and IrNC. **a**, **c**, **d** The isosurface and slices from the top/side-view of the orbitals above the Fermi level for Ir, respectively. **b**, **d**, **f** The isosurface and slices from the top/side-view of the orbitals above the Fermi level for IrNC, respectively

With a weak hydrogen adsorption behavior, Ir was first selected for the hydrogen adsorption/ desorption behavior balancing^16^. As the thermally stable plane of Ir, the (111) plane is most stable and available in the experiment. Thus, the theoretical investigations were conducted on its thermally stable (111) plane. The structures of the calculation models for Ir without/with C/N environment (Ir/IrNC) are shown in Supplementary Figs. 1 and 2. The orbital profiles of Ir and IrNC were analyzed first. Figure 2 shows the different views of the orbitals above the Fermi level for Ir and IrNC, which are predominately composed of empty *d* orbitals for Ir sites. The empty *d* orbitals above the Fermi level in Fig. 2a, b, along with the projected density of states (DOS) distributions of the Ir, C, and N sites (Supplementary Fig. 3), reveal the strong interactions between the Ir and environmental C/N sites. Because of a large wave function overlap, the *p* orbitals of the C/N sites directly interact with the *d*_*z*_^2^ orbitals of the nearby Ir sites. By introducing the C/N environment, the empty *d* orbitals of the surficial Ir sites for IrNC were remarkably reduced, benefiting the activation of surficial Ir sites and electron transport on the Ir surface of IrNC (Fig. 2c, d).

The orbital modulated electron structure of IrNC can be further illustrated using the electron density difference calculation, with Ir as the reference (Supplementary Fig. 4). The electrons of the surficial Ir sites on the Ir(111) surface are more like to transfer to nearby hollow fcc sites. Thus, the hollow fcc sites with high electron density are favored for hydrogen adsorption. Compared with Ir, the electron density of the surficial Ir sites of IrNC is much lower, while it is exactly the opposite for the hollow fcc sites. Therefore, the hollow fcc sites on the surface of IrNC with higher electron density will benefit hydrogen adsorption, leading to enhanced HER performance. Furthermore, the positive shift of the *d*-band center toward the Fermi level (*E*_F_) in IrNC agrees well with the electron density difference results (Supplementary Fig. 5)^17^.

**Fig. 3:**
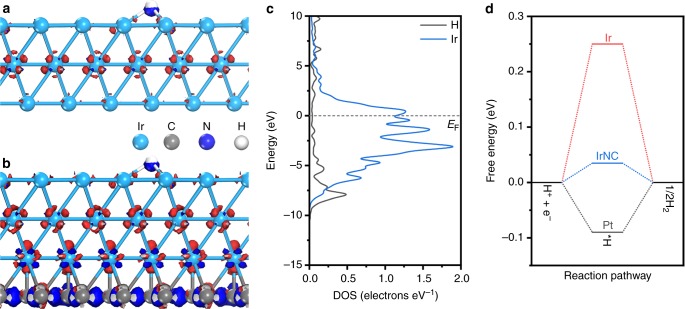
Theoretical calculations of the hydrogen adsorption configured for Ir and IrNC. **a**, **b** The isosurfaces of the electron density difference for Ir and IrNC with H adsorption, respectively. **c** The projected density of states (DOS) distribution of adsorbed H and surficial Ir sites in IrNC, respectively. **d** The calculated free energy diagram of HER on the surface of Ir and IrNC at the equilibrium potential, respectively

To obtain a direct understanding of the hydrogen adsorption/desorption behaviors on the surface of Ir and IrNC, the calculation models were further configured with hydrogen adsorbates. Figure 3a, b shows the optimized configurations and electron density differences of Ir and IrNC with hydrogen adsorbed, respectively. The projected DOS distributions of adsorbed hydrogen and surficial Ir were further analyzed (Fig. 3c and Supplementary Fig. 6). In contrast with Ir, stronger bonding of hydrogen on the surficial Ir sites was obtained for IrNC.

Further calculations were conducted to investigate the overall HER pathway on the surfaces of IrNC, taking Ir and Pt as references (Supplementary Fig. 7). H^+^ + e^−^, adsorbed hydrogen (H*) and 1/2H_2_ were considered as the initial, intermediate and final states of the reaction, respectively. The calculated free energy diagrams of the HER process on the surfaces of Ir, IrNC, and Pt are presented in Fig. 3d. Pt, which is known as the most efficient HER catalyst, exhibits a small upslope of 0.09 eV, revealing hydrogen desorption as the rate-determining step (RDS). Unlike Pt, weak hydrogen adsorption is the RDS of Ir, with a larger upslope of 0.25 eV. Significantly, the hydrogen adsorption/desorption behaviors of surficial Ir sites of the IrNC were largely balanced by the environmental C/N sites, which introduced by orbital modulation, resulting in a much smaller upslope of 0.04 eV. Compared with Pt, the interaction energy between hydrogen and IrNC surface would be not energetically favored for hydrogen adsorption, because of a little positive free energy change in the hydrogen adsorption process. However, this interaction energy would be more energetically favored for hydrogen desorption. The reaction rate of HER depends on the upslope process in the HER free energy diagram. With a much smaller upslope, the interaction energy between hydrogen and IrNC surface would be more favored by the hydrogen adsorption/desorption involved overall HER reaction process, accelerating the overall rate of HER reaction. The above results suggest that IrNC will exhibit strikingly improved catalytic activity toward the hydrogen evolution process, compared with Ir and Pt.

### Catalyst characterizations

Inspired by the theoretical calculation results, hollow nitrogenated carbon nanospheres anchored with IrNP were prepared (IrHNC, Supplementary Figs. 8–10). The structure and crystallinity of the IrHNC were first investigated by field emission scanning electron microscope (Supplementary Fig. 11a) and high-power X-ray diffraction (HP-XRD, Supplementary Figs. 11b and 12). The broad peak at around 23° belongs to the carbon layer of the nitrogenated carbon with low crystallinity, while the weak peaks at around 40.6°, 47.3°, and 69.1° can be attributed to the (111), (200), and (220) planes of cubic Ir. Meanwhile, according to the Debye–Scherrer equation, an average particle size of IrNP is around 2.1 nm.

**Fig. 4:**
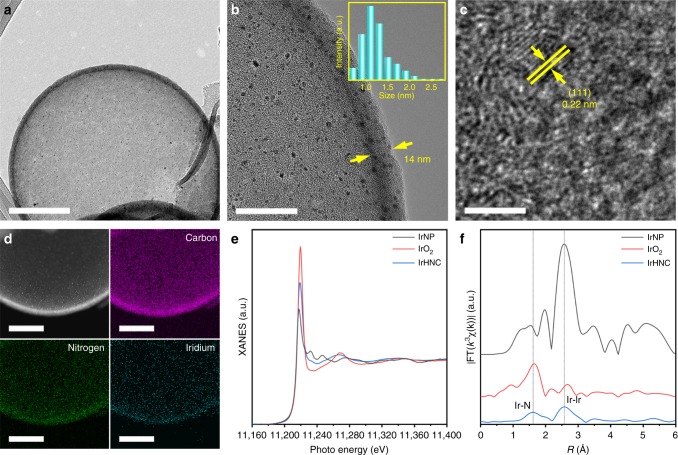
Structural characterization of IrHNC. **a**, **b** Low-resolution TEM images of IrHNC. Inset is the corresponding particle size distribution of the Ir nanoparticles. **c** High-resolution TEM image of IrHNC. **d** Elemental mapping (purple: carbon, green: nitrogen, cyanic blue: iridium). **e** Ir L_III_-edge XANES spectra of IrNP, IrO_2_, and IrHNC, respectively. **f** Fourier transform (FT) of the Ir L_III_-edge EXAFS spectra of IrNP, IrO_2_, and IrHNC, respectively. Scale bars: **a** 200 nm, **b** 40 nm, **c** 3 nm, **d** 100 nm

The detailed morphology of the IrHNC was further studied on a high-resolution transmission electron microscope (HR-TEM, Fig. 4). The size of a building block in the IrHNC is around 700 nm (Fig. 4a). The hollow nanosphere had a porous structured shell with a thickness of about 14 nm. The size distribution of IrNP was in the range of 0.9–2.5 nm and they were uniformly anchored in the porous shell (Fig. 4b). The lattice fringes in the HR-TEM image exhibited a *d*-spacing of 0.22 nm, which can be assigned to the {111} facets of cubic Ir (Fig. 4c). The homogeneous distribution of the C, N, and Ir elements was further revealed by the high-angle annular dark-field scanning transmission electron microscopy and element mapping images (Fig. 4d).

The analysis of the chemical composition of the IrHNC was conducted using X-ray photoelectron spectroscopy (XPS, Supplementary Fig. 13). The concentration of N species, mainly composed of pyridinic, graphitic, and oxidized N species, was around 4.45 at%^18^. The peak at 61.4 eV belongs to the metallic Ir°, which exhibited a low concentration of 0.36 at% (Supplementary Table 1). Compared with the IrNPs, a slight blueshift was observed for IrHNC, which further suggests a strong interaction between the Ir and N species (Supplementary Figs. 14 and 15)^15^. Highly sensitive X-ray absorption fine structure (XAFS) tests based on synchrotron radiation were performed to obtain deeper insight of the structural nature of the IrHNC. With a +4 oxidation state, the position of the white line in the Ir L_III_-edge X-ray absorption near-edge structure (XANES) spectra of IrO_2_ was more positive than those of IrNP and IrHNC (Fig. 4e). Compared with the 0 oxidation state of IrNP, that of IrHNC exhibits a small positive shift, in agreement with the above XPS analysis. The white line features can be confirmed more clearly in the second derivatives of the Ir L_III_-edge XANES spectra (Supplementary Fig. 16). The corresponding Fourier-transformed (FT) k^3^-weighted extended XAFS (EXAFSs) are shown in Fig. 4f. The main peaks in the EXAFS spectra of the IrNP and IrHNC at around 2.58 Å belong to the Ir–Ir coordination. Moreover, an Ir–N coordination peak at around 1.59 Å appears in the EXAFS spectrum of IrHNC, confirming the interaction between the Ir and N species.

A thermogravimetric analysis (TGA) further confirmed the Ir content of the IrHNC to be as low as 7.16 wt% (Supplementary Fig. 17). According to the nitrogen adsorption–desorption isotherms, the IrHNC possessed a specific surface area of 759.4 m^2^ g^−1^ and a pore volume of 0.76 cm^3^ g^−1^, respectively (Supplementary Fig. 18).

### Catalyst performance

**Fig. 5:**
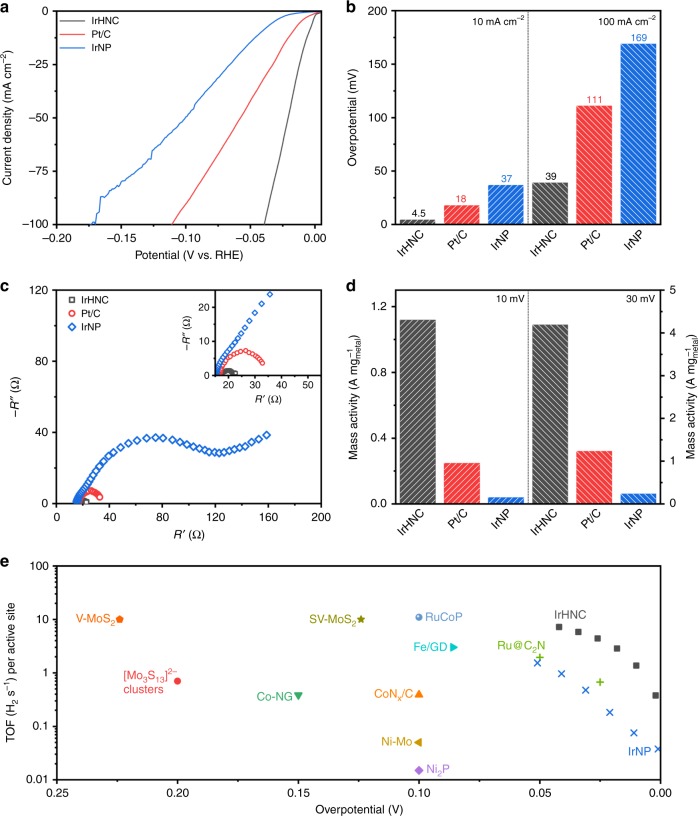
Electrochemical performance of IrHNC. **a** Polarization curves IrHNC, Pt/C, and IrNP in nitrogen saturated 0.5 M aq. H_2_SO_4_ solution. **b** Overpotentials of IrHNC, Pt/C and IrNP at current densities of 10 and 100 mA cm^−2^. **c** Electrochemical impedance spectroscopy (EIS) curves of IrHNC, Pt/C and IrNP at an overpotential of 10 mV. **d** Mass activities of IrHNC, Pt/C, and IrNP at overpotentials of 10 and 30 mV. **e** The TOF values of IrHNC and other recently reported HER electrocatalysts in acidic media. Scan rate: 5 mV s^−1^

To verify the theoretical prediction, the catalytic HER performance of the obtained IrHNC was evaluated in nitrogen saturated 0.5 M aq. H_2_SO_4_. IrNP and commercial Pt/C were also investigated as references (Supplementary Figs. 19 and 20). Figure 5a shows the polarization curves of the IrHNC, IrNP, and Pt/C. Remarkably, with the same concentration of metal, IrHNC exhibited significantly enhanced HER performance. To achieve a current density of 10 and 100 mA cm^−2^, the IrHNC only needed small overpotentials of 4.5 and 39 mV, while those for IrNP and Pt/C are much larger (Fig. 5b). These values are also much smaller than those reported for efficient HER catalysts, revealing the superior catalytic activity of IrHNC (Supplementary Table 2)^6,19–22^.

The charge transfer resistance (*R*_ct_) during the hydrogen evolution process was further studied by electrochemical impedance spectroscopy. At an overpotential of 10 mV, IrHNC exhibited the smallest *R*_ct_ of 4.2 Ω, which confirms that the fastest HER reaction kinetics occurred on the surface of the IrHNC (Fig. 5c, Supplementary Fig. 19c).

Mass activity and intrinsic activity are two important parameters to evaluate catalysts for potential practical applications. The mass activities of IrHC, IrNP, and Pt/C at an overpotential of 10 mV are 1.12, 0.04, and 0.25 A mg_metal_^−1^, respectively (Fig. 5d). Remarkably, that value for IrHNC is over 28 and 4.5 times higher than those for IrNP and Pt/C, suggesting its higher potential for practical applications.

The turnover frequencies (TOFs) of the IrHNC and IrNP were further studied to obtain deeper insight into their intrinsic activities, using the reported method^23^. As shown in Fig. 5e, the TOFs of the IrHNC and IrNP were around 4.21 and 0.28 H_2_ s^−1^ at 25 mV, respectively. Interestingly, the value for IrHNC is over 15 times higher than that for IrNP, indicating the IrHNC has significantly enhanced intrinsic activity. Moreover, this value is also far superior to other reported efficient HER electrocatalysts, including Ru@C_2_N, RuCoP, Fe/GD, CoN_*x*_/C, Ni–Mo, Ni_2_P, and Co–NG^8,23–30^. More importantly, the IrHNC also exhibited a Faradaic efficiency as high as about 100% (Supplementary Fig. 19e).

The greatly improved reaction kinetics and intrinsic activity of IrHNC toward the hydrogen evolution catalysis are also consistent with the theoretical predictions.

## Discussion

In summary, we report the rational design of an efficient catalyst for hydrogen evolution, by balancing hydrogen adsorption/desorption via orbital modulation. Theoretical calculations suggested that the *d* orbitals of Ir sites can be manipulated through strong interactions with the *p* orbitals of C/N atoms. They suggested that orbital modulation would further balance the hydrogen adsorption/desorption behaviors of the surficial Ir sites, enabling efficient hydrogen evolution. Inspired by the theoretical results, IrHNC was prepared by anchoring a low content of IrNP on nitrogenated carbon matrixes. As expected, the IrHNC exhibited significantly enhanced reaction kinetics, mass activity and intrinsic activity for the hydrogen evolution. This work not only highlights the rational design of a catalyst for efficient hydrogen evolution, but also introduces an opportunity to achieve enhanced catalytic performance for diverse reactions via orbital modulation.

## Methods

### Theoretical calculation

The first principle DFT calculations were performed using the Vienna Ab Initio Simulation Package. The Perdew–Burke–Ernzerhof (PBE) type gradient-corrected exchange-correlation potential can provide a better overall description of the electronic subsystem, which is especially suitable for the studies of molecular interaction with metal surfaces^31^. Meanwhile, van der Waals interactions have been usually considered for the combined system^32^. Thus, the PBE type gradient-corrected exchange-correlation potential was employed in this study, considering van der Waals interactions. Projector-augmented wave potentials were used to describe the ionic potentials. A cutoff energy of 400 eV was used for the plane-wave basis set. A 4 × 4 × 1 Monkhorst–Pack k-point setup was used, and the atomic configurations were relaxed, with residual forces smaller than 0.01 eV/Å. A vacuum thickness of around 15 Å was set along the z-direction to avoid interaction between periodic images^33,34^.

### Synthesis of IrHNC

As a typical synthesis, 2-amino-2-(hydroxylmethyl)-1,3-propanediol (Tris base, 0.169 g) was dissolved in deionized water (130 ml) with polystyrene (PS) microsphere (750 nm, 0.5 g), forming solution A. Dopamine hydrochloride (0.5 g) and IrCl_3_·xH_2_O (15 mg) were dissolved in deionized water (10 ml), forming solution B. The above solutions were stirred for 1 h at ambient conditions. Solution B was quickly poured into solution A, and stirred for another 26 h. The product was collected by centrifuge and dried in an oven under reduced pressure, then designated PS@IrPDA. The PS@IrPDA was annealed at 800 °C for 2 h in an argon atmosphere, denoted as IrHNC. IrNP were prepared following the reported liquid phase reduction method^19^, and designated IrNP.

### Materials characterization

The morphologies of the samples were characterized on a field emission scanning electron microscope (SEM, Nanonova 230, FEI, USA) and high-resolution transmission electron microscopy (HR-TEM, JEM-2100F, JEOL, Japan). XRD patterns were obtained from a high-power X-ray diffractometer (D/MAZX 2500V/PC, Rigaku, Japan). The XPS test was conducted using an X-ray photoelectron spectrometer (K-alpha, Thermo Fisher Scientific, UK). The XAFS test was performed at the 6D UNIST-PAL beam-line of Pohang Light Source (PLS-II) in Korea. The specific surface area was analyzed using nitrogen adsorption–desorption isotherms, and the Brunauer-Emmett-Teller (BET) method (BELSORP-max, BEL, Japan). The TGA was conducted on a Thermogravimetric Analyzer (Q200, TA, USA).

### Electrochemical characterizations

The electrocatalytic hydrogen evolution performance was evaluated using an electrochemical workstation (Ivium, Netherlands) with a typical three-electrode cell. To prevent Pt contamination, a graphite rod was used as the counter-electrode. An Ag/AgCl (3 M KCl) electrode was used as a reference electrode. All the potentials were referenced *vs*. RHE. To form uniform catalyst inks, the catalysts and Nafion (5 wt% in a mixture of lower aliphatic alcohol and water, Aldrich Chemical Inc.) were dispersed in an isopropyl alcohol solution by ultrasonication. The inks were dropped onto a glassy carbon electrode (3 mm in diameter) to form a film for the electrochemical tests. The loading amounts for IrHNC, IrNP, and Pt/C were 18 and 18 μg_Pt_ cm^−2^, respectively. Linear sweep voltammetry was conducted in a nitrogen saturated 0.5 M aq. H_2_SO_4_ solution at a scan rate of 5 mV s^*−*1^. Electrochemical impedance spectroscopy measurements were conducted over a frequency range of 0.1–10,000 Hz at overpotentials of 10 mV. All the data presented were corrected with solution resistance.

## Supplementary information

Supplementary Information

## Data Availability

The data that supports the findings of this study are available from the corresponding author upon reasonable request.

## References

[1] Turner JA (2004). Sustainable hydrogen production. Science.

[2] Norskov JK, Christensen CH (2006). Chemistry—toward efficient hydrogen production at surfaces. Science.

[3] Cook TR (2010). Solar energy supply and storage for the legacy and non legacy worlds. Chem. Rev..

[4] Voiry D (2013). Enhanced catalytic activity in strained chemically exfoliated WS_2_ nanosheets for hydrogen evolution. Nat. Mater..

[5] Morales-Guio CG, Stern LA, Hu XL (2014). Nanostructured hydrotreating catalysts for electrochemical hydrogen evolution. Chem. Soc. Rev..

[6] Yin HJ (2015). Ultrathin platinum nanowires grown on single-layered nickel hydroxide with high hydrogen evolution activity. Nat. Commun..

[7] Zou XX, Zhang Y (2015). Noble metal-free hydrogen evolution catalysts for water splitting. Chem. Soc. Rev..

[8] Li H (2016). Activating and optimizing MoS_2_ basal planes for hydrogen evolution through the formation of strained sulphur vacancies. Nat. Mater..

[9] Wang XS (2018). Strain effect in bimetallic electrocatalysts in the hydrogen evolution reaction. ACS Energy Lett..

[10] Shan JQ (2019). Charge-redistribution-enhanced nanocrystalline Ru@IrO_x_ electrocatalysts for oxygen evolution in acidic media. Chemistry.

[11] Shan JQ, Ling T, Davey K, Zheng Y, Qiao SZ (2019). Transition-metal-doped RuIr bifunctional nanocrystals for overall water splitting in acidic environments. Adv. Mater..

[12] Hinnemann B (2005). Biomimetic hydrogen evolution: MoS_2_ nanoparticles as catalyst for hydrogen evolution. J. Am. Chem. Soc..

[13] Subbaraman R (2011). Enhancing hydrogen evolution activity in water splitting by tailoring Li^+^-Ni(OH)_2_-Pt interfaces. Science.

[14] Gong M (2014). Nanoscale nickel oxide/nickel heterostructures for active hydrogen evolution electrocatalysis. Nat. Commun..

[15] Wu YS (2018). Electron density modulation of NiCo_2_S_4_ nanowires by nitrogen incorporation for highly efficient hydrogen evolution catalysis. Nat. Commun..

[16] Engstrom JR, Tsai W, Weinberg WH (1987). The chemisorption of hydrogen on the (111) and (110)-(1x2) surfaces of iridium and platinum. J. Chem. Phys..

[17] Chen ZY (2018). Tailoring the d-band centers enables Co_4_N nanosheets to be highly active for hydrogen evolution catalysis. Angew. Chem. Int. Ed..

[18] Li F (2018). Boosting oxygen reduction catalysis with abundant copper single atom active sites. Energ. Environ. Sci..

[19] Huang X (2013). Solution-phase epitaxial growth of noble metal nanostructures on dispersible single-layer molybdenum disulfide nanosheets. Nat. Commun..

[20] Zhu LL (2016). A rhodium/silicon co-electrocatalyst design concept to surpass platinum hydrogen evolution activity at high overpotentials. Nat. Commun..

[21] Wang PT (2017). Precise tuning in platinum-nickel/nickel sulfide interface nanowires for synergistic hydrogen evolution catalysis. Nat. Commun..

[22] Li F (2018). Mechanochemically assisted synthesis of a Ru catalyst for hydrogen evolution with performance superior to Pt in both acidic and alkaline media. Adv. Mater..

[23] Kibsgaard J, Jaramillo TF, Besenbacher F (2014). Building an appropriate active-site motif into a hydrogen-evolution catalyst with thiomolybdate [Mo_3_S_13_]^2-^ clusters. Nat. Chem..

[24] McKone JR, Sadtler BF, Werlang CA, Lewis NS, Gray HB (2013). Ni-Mo nanopowders for efficient electrochemical hydrogen evolution. ACS Catal..

[25] Popczun EJ (2013). Nanostructured nickel phosphide as an electrocatalyst for the hydrogen evolution reaction. J. Am. Chem. Soc..

[26] Fei HL (2015). Atomic cobalt on nitrogen-doped graphene for hydrogen generation. Nat. Commun..

[27] Liang HW (2015). Molecular metal-N_x_ centres in porous carbon for electrocatalytic hydrogen evolution. Nat. Commun..

[28] Mahmood J (2017). An efficient and pH-universal ruthenium-based catalyst for the hydrogen evolution reaction. Nat. Nanotechnol..

[29] Xu JY (2018). Boosting the hydrogen evolution performance of ruthenium clusters through synergistic coupling with cobalt phosphide. Energy Environ. Sci..

[30] Xue YR (2018). Anchoring zero valence single atoms of nickel and iron on graphdiyne for hydrogen evolution. Nat. Commun..

[31] Perdew JP, Burke K, Ernzerhof M (1996). Generalized gradient approximation made simple. Phys. Rev. Lett..

[32] Tkatchenko A, Scheffler M (2009). Accurate molecular van der Waals interactions from ground-state electron density and free-atom reference data. Phys. Rev. Lett..

[33] Hammer B, Hansen LB, Norskov JK (1999). Improved adsorption energetics within density-functional theory using revised Perdew-Burke-Ernzerhof functionals. Phys. Rev. B.

[34] Helveg S (2004). Atomic-scale imaging of carbon nanofibre growth. Nature.

