# Supplementary material for: Balancing hydrogen adsorption/desorption by orbital modulation for efficient hydrogen evolution catalysis
Source: Nat Commun. 2019 Sep 6;10:4060. doi: 10.1038/s41467-019-12012-z (PMC6731251; doi:10.1038/s41467-019-12012-z)
Supplement: Supplementary file 1 — Supplementary Information [file 41467_2019_12012_MOESM1_ESM.pdf]

**Supplementary Information**

# Balancing hydrogen adsorption/desorption by orbital modulation for efficient hydrogen evolution catalysis

By Li *et al*

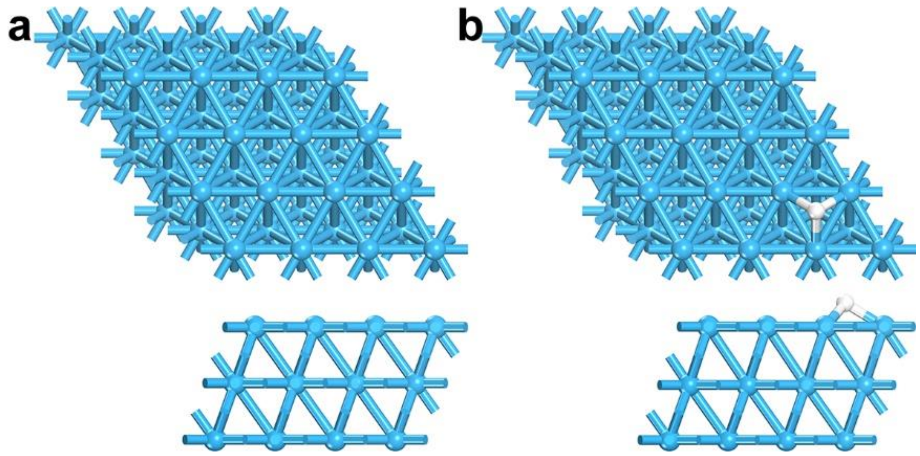


**Supplementary Figure 1 | a**, **b**, The top-view and side-view optimized structures of Ir and H adsorbed Ir. Sky blue and white ivory balls represent iridium and hydrogen atoms, respectively.

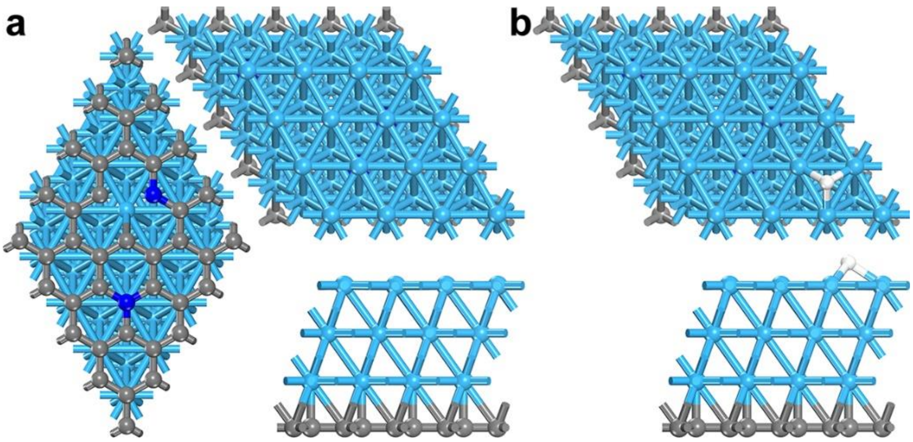


**Supplementary Figure 2 | a**, **b**, The top-view and side-view optimized structures of IrNC and H adsorbed IrNC. Grey, blue, sky blue and white ivory balls represent carbon, nitrogen, iridium and hydrogen atoms, respectively.

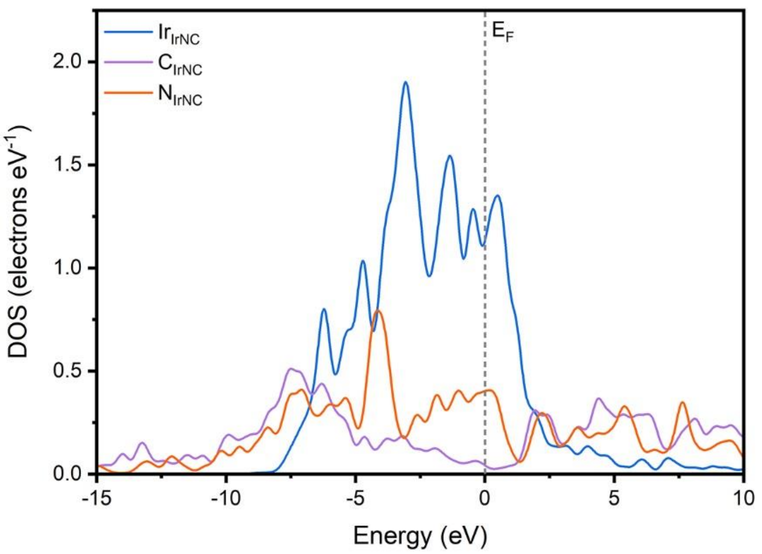


**Supplementary Figure 3 |** The projected density of states (DOS) distribution of Ir, C and N in IrNC, respectively.

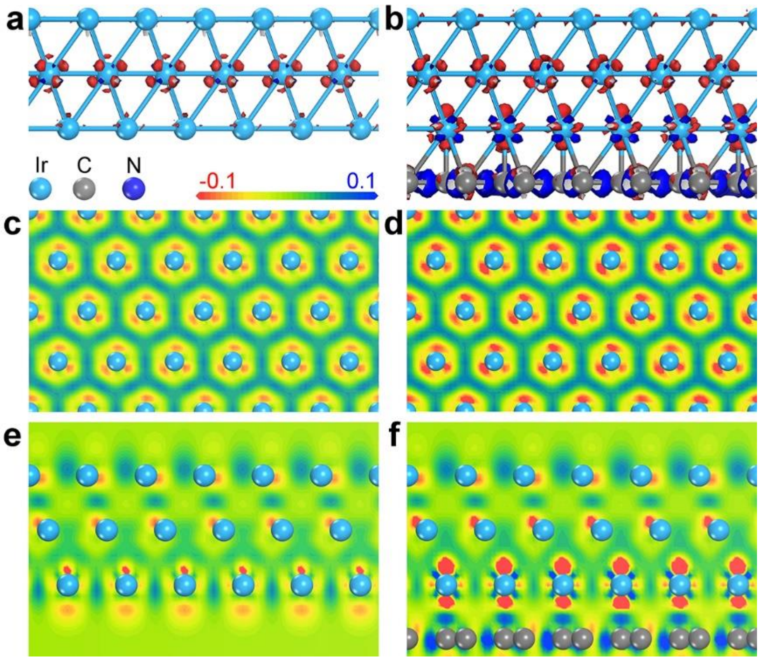


**Supplementary Figure 4 | Theoretical calculations of the electron density differences of Ir and IrNC. a**,**c**,**d**, The isosurface, and slices from the top-/side-view of the electron density difference for Ir, respectively. **b**,**d**,**f**, The isosurface, and slices from the top-/side-view of the electron density difference for IrNC, respectively. The red isosurfaces denote an increase of 0.1 e $Å^{-3}$ in electronic density, while the blue isosurfaces denote a decrease of 0.1 e $Å^{-3}$.

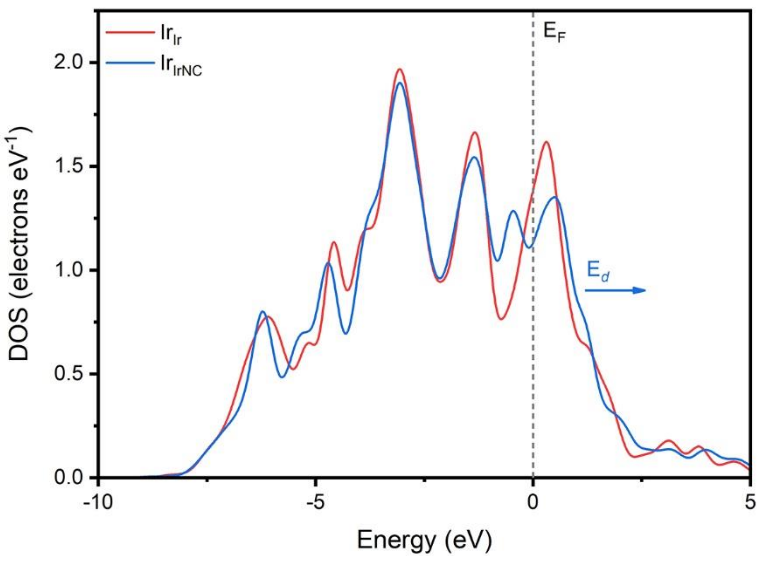


**Supplementary Figure 5 |** The projected density of states (DOS) distribution of surficial Ir in Ir and IrNC, respectively. The corresponding d-band centres ($E_d$) are −1.78 and −1.71 eV, respectively.

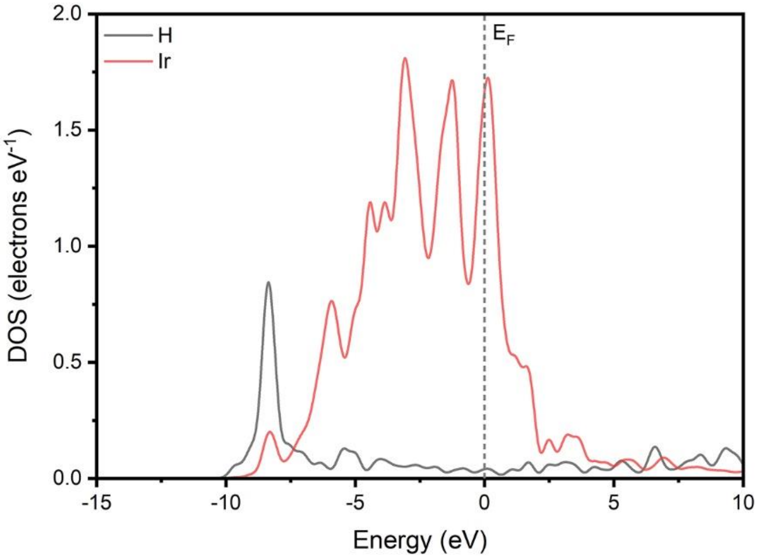


**Supplementary Figure 6 |** The projected DOS distribution of adsorbed H and surficial Ir sites in Ir, respectively.

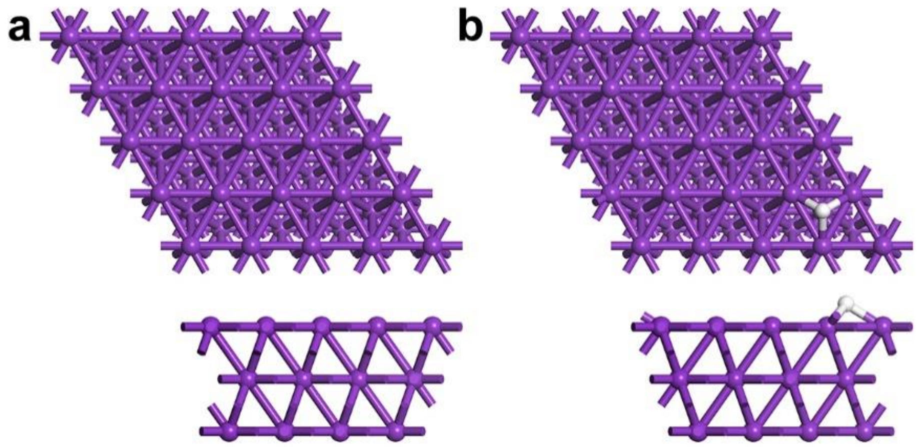


**Supplementary Figure 7 | a**, **b**, The top-view and side-view optimized structures of Pt and H adsorbed Pt. Purple and white ivory balls represent platinum and hydrogen atoms, respectively.

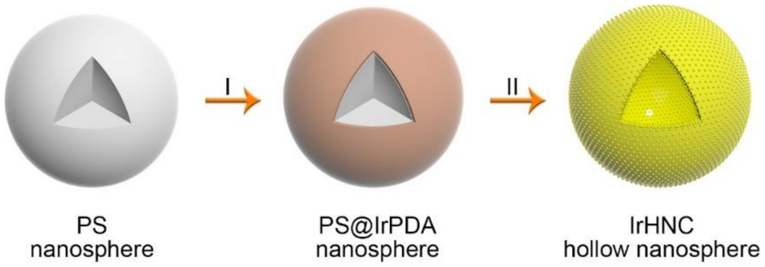


**Supplementary Figure 8 |** Schematic illustration of the synthesis of IrHNC. (I) polymerization of Ir coordinated dopamine on the surface of a PS nanosphere; (II) Pyrolysis of PS@IrPDA nanosphere at high temperature.

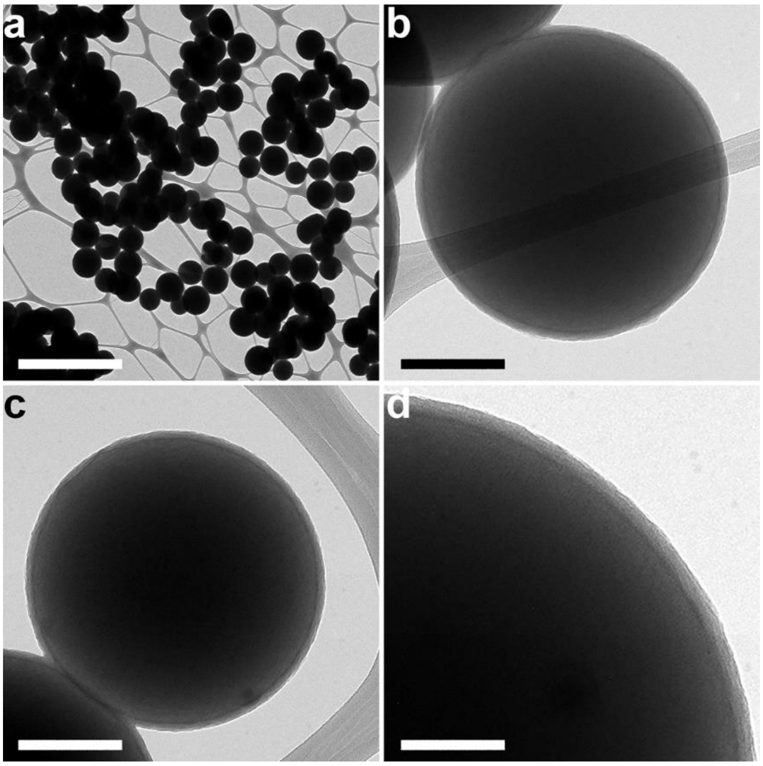


**Supplementary Figure 9 │ a-c**, Low resolution TEM images, **d**, high resolution TEM image of PS@IrPDA nanospheres. Scale bars: **a**, 3 μm; **b**, **c**, 200 nm; **d**, 100 nm.

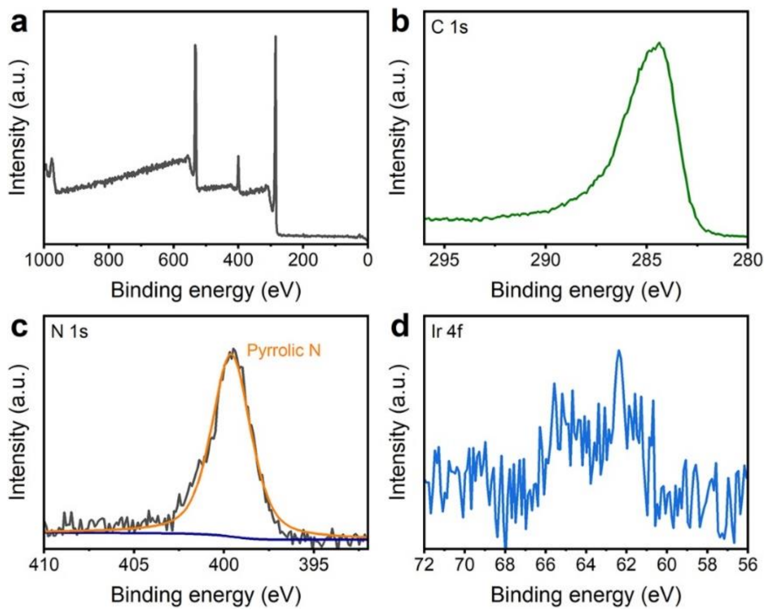


**Supplementary Figure 10 | a**, Full survey XPS spectrum of PS@IrPDA. **b**, High-resolution C 1s spectrum of PS@IrPDA. **c**, High-resolution N 1s spectrum of PS@IrPDA. **d**, High-resolution Ir 4f spectrum of PS@IrPDA.

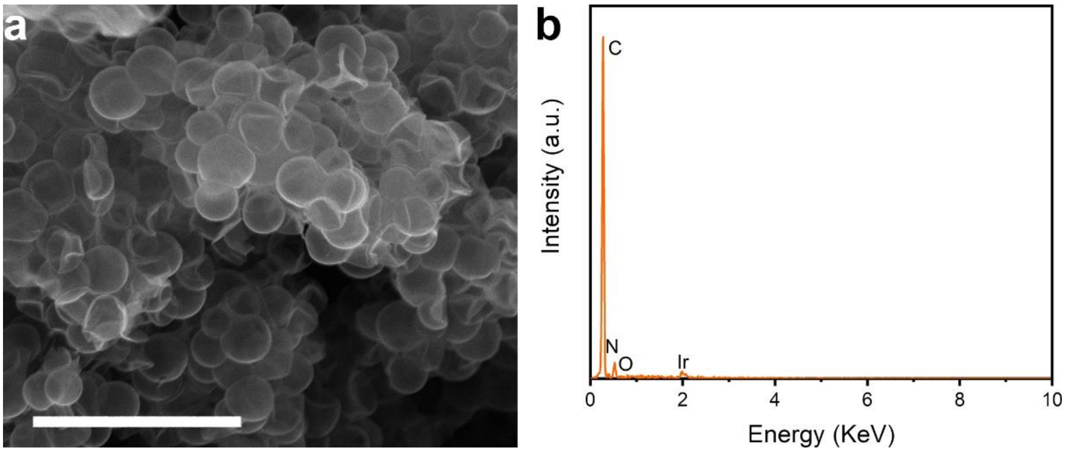


**Supplementary Figure 11 | a**, Low resolution SEM images of IrHNC. **b**, SEM-EDS spectrum of IrHNC. Scale bar: **a**, 3 μm.

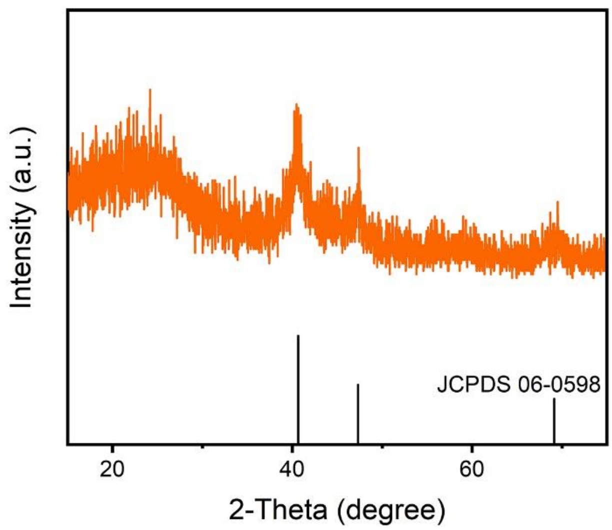


**Supplementary Figure 12 |** XRD patterns for IrHNC and metallic Ir (JCPDS no. 06-0598).

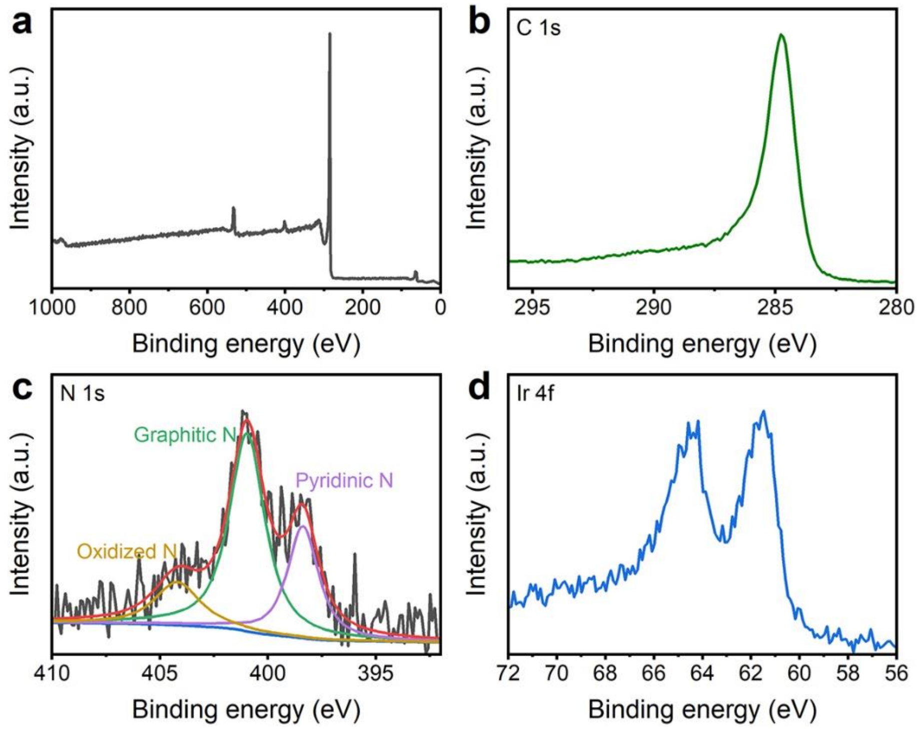


**Supplementary Figure 13 | a**, Full survey XPS spectrum of IrHNC. **b**, High-resolution C 1s spectrum of IrHNC. **c**, High-resolution N 1s spectrum of IrHNC. **d**, High-resolution Ir 4f spectrum of IrHNC.

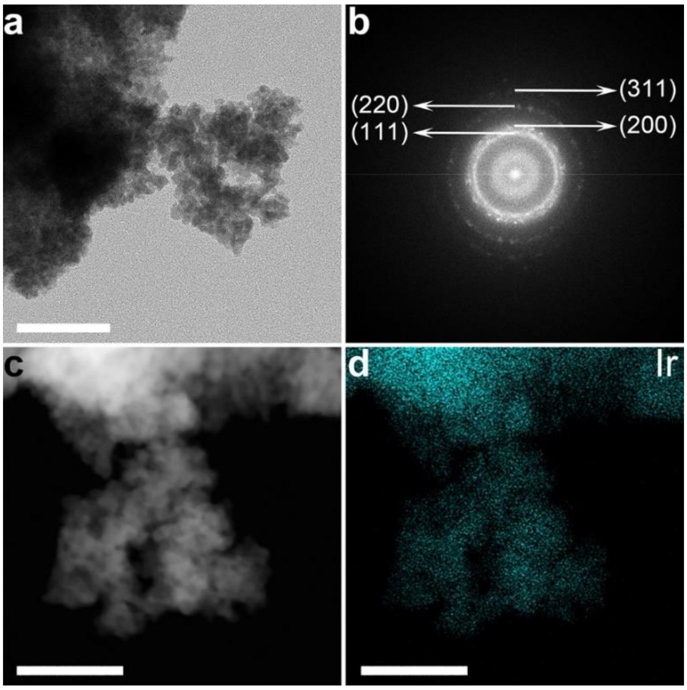


**Supplementary Figure 14 | a**, High resolution TEM image of IrNP. The average size of the Ir nanoparticles is less than 2 nm. **b**, Corresponding FFT pattern. The indexed crystal facets belong to metallic Ir (JCPDS no. 06-0598). **c**, **d**, The HAADF-STEM and elemental mapping images of IrNP. (cyanic blue: iridium). Scale bars: **a**, 30 nm; **c-d**, 25 nm.

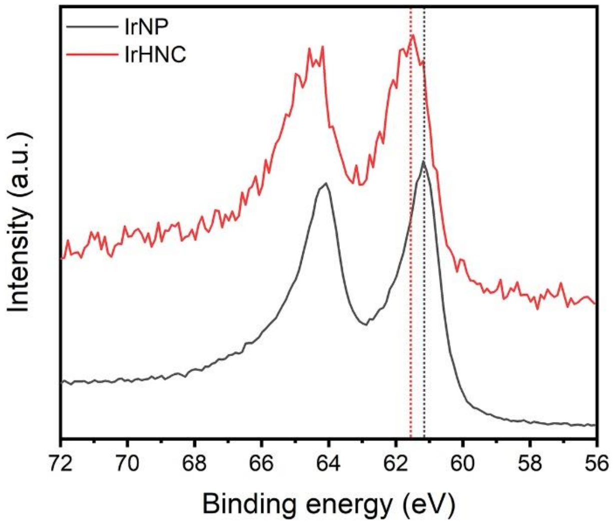


**Supplementary Figure 15 |** XPS Ir 4f profiles of IrHNC and IrNP. Compared with IrNP, the Ir $4f_{7/2}$ peak for IrHNC exhibits a slight blueshift, confirming the strong interaction between the Ir and N.

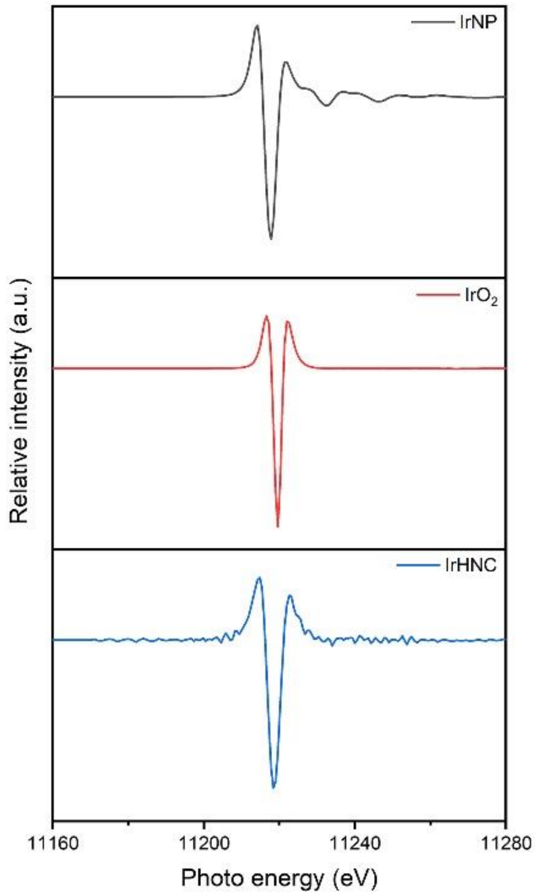


**Supplementary Figure 16 |** Second derivatives of XANES spectra of IrNP, $IrO_2$ and IrHNC, respectively.

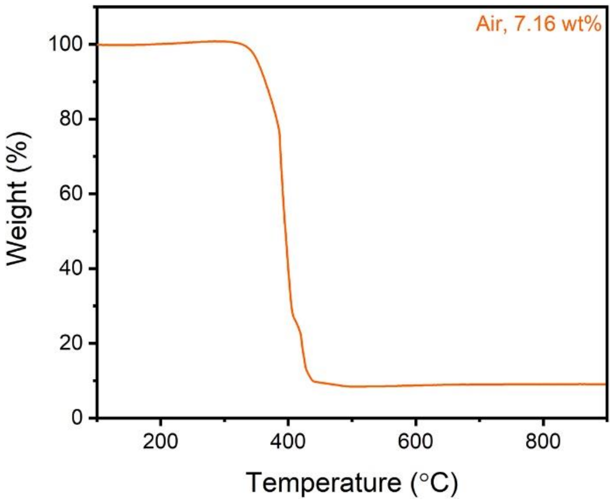


**Supplementary Figure 17 | **TGA curve of IrHNC under air atmosphere with a ramping rate of 10 °C $min^{-1}$.

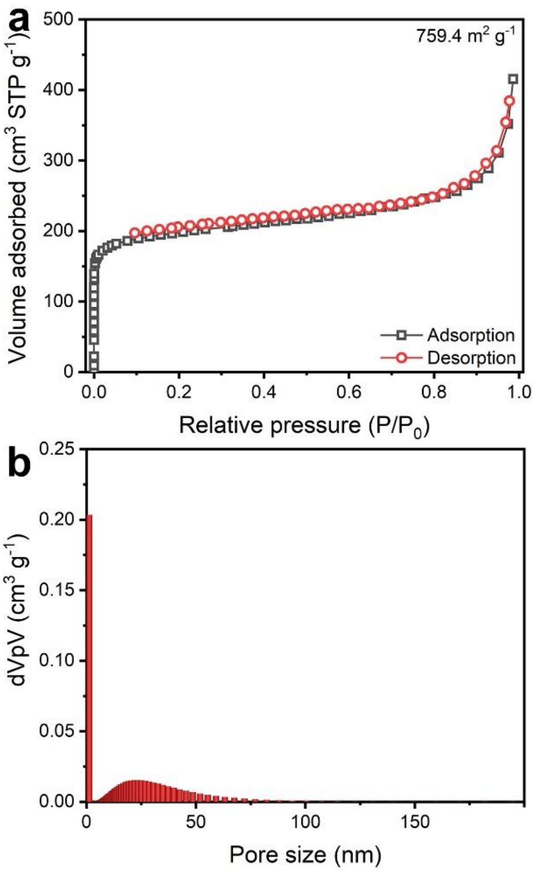


**Supplementary Figure 18 | a**, $N_2$ adsorption/desorption isotherms of IrHNC. **b**, Corresponding pore distribution. The specific surface area is around 759.4 $m^2$ $g^{-1}$.

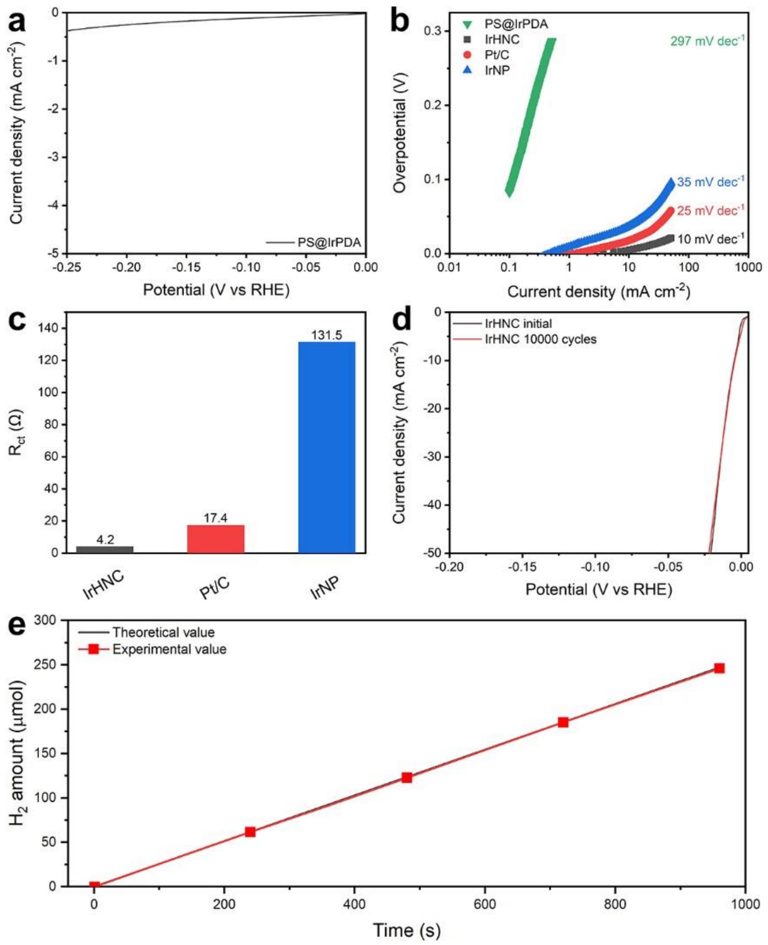


**Supplementary Figure 19 | a**, Polarization curves PS@IrPDA in nitrogen saturated 0.5 M *aq.* $H_2SO_4$ solution. **b**, Corresponding Tafel plots of PS@IrPDA, IrHNC, Pt/C and IrNP. **c**, $R_{ct}$s of IrHNC, Pt/C and IrNP at 10 mV (*vs.* RHE), respectively. **d**, Stability test of IrHNC in 0.5 M *aq.* $H_2SO_4$ solution. The polarization curves were recorded before and after 10000 potential cycles from 0.1 to −0.2 V (*vs.* RHE). **e**, Hydrogen production over IrHNC at a current density of 20 mA cm$^{-2}$. The Faradaic efficiency is around 100%.

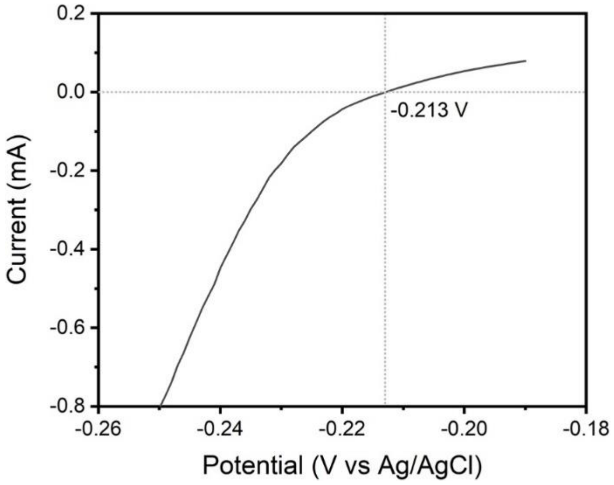


**Supplementary Figure 20 |** Calibration of the Ag/AgCl electrode with respect to RHE, using the polarization curve of Pt black in highly pure $H_2$ saturated 0.5 M *aq.* $H_2SO_4$ solution. Scan rate: 1 mV $s^{-1}$.

**Supplementary Table 1 |** Element quantification of PS@IrPDA and IrHNC from XPS

| Sample | C (at%) | O (at%) | N (at%) | Ir (at%) |
|---|---|---|---|---|
| **PS@IrPDA** | 72.61 | 20.19 | 7.20 | Too low to quantify |
| **IrHNC** | 89.98 | 5.21 | 4.45 | 0.36 |

**Supplementary Table 2 |** HER parameters for recently reported efficient catalysts in acidic electrolyte

| Catalyst | Catalyst loading amount | Current density (mA $cm^{-2}$) | Overpotential at corresponding j (mV) | Reference |
|---|---|---|---|---|
| **IrHNC** | 0.25 mg $cm^{-2}$<br>18 $\mu g_{Ir}$ $cm^{-2}$ | 10<br>20<br>100 | 4.5<br>10<br>39 | This work |
| **$Ru@C_2N$** | 0.285 mg $cm^{-2}$ | 10<br>20 | 22<br>34.8 | 1 |
| **Rh/SiNW** | 0.193 mg $cm^{-2}$<br>56 $\mu g_{Rh}$ $cm^{-2}$ | 10<br>100 | ~84<br>180 | 2 |
| **$Pt_3Ni_2$ NWs-S/C** | 15.3$\mu g_{Pt}$ $cm^{-2}$ | 10 | ~25 | 3 |
| **$[Mo_3S_{13}]^{2-}$ clusters** | 0.1 mg $cm^{-2}$ | 10 | 180 | 4 |
| **CoPS nanoplate** | -- | 10 | 48 | 5 |
| **SV-$MoS_2$** | -- | 10 | 170 | 6 |
| **Exfoliated $WS_2$ nanosheets** | 6.5 µg $cm^{-2}$ | 10 | 234 | 7 |
| **$CoMoS_x$** | 50 µg $cm^{-2}$ | 5 | 207 | 8 |
| **A-Ni–C** | 0.283 mg $cm^{-2}$ | 10<br>20 | 34<br>48 | 9 |
| **CoS\|P/CNT** | 1.6 mg $cm^{-2}$ | 10 | 64 | 10 |
| **$WO_{2.9}$** | 0.285 mg $cm^{-2}$ | 10 | 70 | 11 |
| **$MoS_2/CoSe_2$** | 0.28 mg $cm^{-2}$ | 10 | 68 | 12 |
| **$CoN_x/C$** | 2 mg $cm^{-2}$ | 10 | 133 | 13 |
| **Edgeterminated $MoS_2$** | 0.28 mg $cm^{-2}$ | 10 | 149 | 14 |
| **Pt NWs/SLNi$(OH)_2$** | -- | 5 | 95 | 15 |
| **Pt-$MoS_2$** | 75 µg $cm^{-2}$ | 10 | 53 | 16 |

## Supplementary References


1. Mahmood, J. et al. An efficient and pH-universal ruthenium-based catalyst for the hydrogen evolution reaction. *Nat. Nanotechnol.* **12**, 441-446 (2017).

2. Zhu, L. L. et al. A rhodium/silicon co-electrocatalyst design concept to surpass platinum hydrogen evolution activity at high overpotentials. *Nat. Commun.* **7**, 12272 (2016).

3. Wang, P. T. et al. Precise tuning in platinum-nickel/nickel sulfide interface nanowires for synergistic hydrogen evolution catalysis. *Nat. Commun.* **8**, 14580 (2017).

4. Kibsgaard, J., Jaramillo, T. F. & Besenbacher, F. Building an appropriate active-site motif into a hydrogen-evolution catalyst with thiomolybdate $[Mo_3S_{13}]^{2-}$ clusters. *Nat. Chem.* **6**, 248-253 (2014).

5. Caban-Acevedo, M. et al. Efficient hydrogen evolution catalysis using ternary pyrite-type cobalt phosphosulphide. *Nat. Mater.* **14**, 1245-1251 (2015).

6. Li, H. et al. Activating and optimizing $MoS_2$ basal planes for hydrogen evolution through the formation of strained sulphur vacancies. *Nat. Mater.* **15**, 48-53 (2016).

7. Voiry, D. et al. Enhanced catalytic activity in strained chemically exfoliated $WS_2$ nanosheets for hydrogen evolution. *Nat. Mater.* **12**, 850-855 (2013).

8. Staszak-Jirkovsky, J. et al. Design of active and stable Co-Mo-$S_x$ chalcogels as pH-universal catalysts for the hydrogen evolution reaction. *Nat. Mater.* **15**, 197-203 (2016).

9. Fan, L. L. et al. Atomically isolated nickel species anchored on graphitized carbon for efficient hydrogen evolution electrocatalysis. *Nat. Commun.* **7**, 10667 (2016).

10. Liu, W. et al. A highly active and stable hydrogen evolution catalyst based on pyrite-structured cobalt phosphosulfide. *Nat. Commun.* **7**, 10771 (2016).

11. Li, Y. H. et al. Local atomic structure modulations activate metal oxide as electrocatalyst for hydrogen evolution in acidic water. *Nat. Commun.* **6**, 8064 (2015).

12. Gao, M. R. et al. An efficient molybdenum disulfide/cobalt diselenide hybrid catalyst for electrochemical hydrogen generation. *Nat. Commun.* **6**, 5982 (2015).

13. Liang, H. W. et al. Molecular metal-$N_x$ centres in porous carbon for electrocatalytic hydrogen evolution. *Nat. Commun.* **6**, 7992 (2015).

14. Gao, M. R., Chan, M. K. Y. & Sun, Y. G. Edge-terminated molybdenum disulfide with a 9.4-Å interlayer spacing for electrochemical hydrogen production. *Nat. Commun.* **6**, 7493 (2015).

15. Yin, H. J. et al. Ultrathin platinum nanowires grown on single-layered nickel hydroxide with high hydrogen evolution activity. *Nat. Commun.* **6**, 6430 (2015).

16. Huang, X. et al. Solution-phase epitaxial growth of noble metal nanostructures on dispersible single-layer molybdenum disulfide nanosheets. *Nat. Commun.* **4**, 1444 (2013).
